# CAN-SAR: A database of Canadian species at risk information

**DOI:** 10.1038/s41597-022-01381-8

**Published:** 2022-06-09

**Authors:** Ilona Naujokaitis-Lewis, Sarah Endicott, Jessica M. Guezen

**Affiliations:** 1 National Wildlife Research Centre, Environment and Climate Change Canada, 1125 Colonel by Drive, Ottawa, ON K1S 5B6 Canada; 2 Department of Geography, Carleton University, Ottawa, ON K1S 5B6 Canada; 3 School of Environmental Sciences, University of Guelph, Guelph, ON N1G 2W1 Canada

**Keywords:** Conservation biology, Conservation biology, Biodiversity, Environmental impact

## Abstract

Threatened species lists describe the conservation status of species and are key tools used to inform decisions for biodiversity conservation. These lists are rich in information obtained during status assessment and recovery planning processes, ranging from biological attributes to actions that support recovery. Data compiled from species lists allow for analyses, including assessing trends in threats, prioritizing actions, and identifying barriers to achieving recovery objectives. For legally protected species at risk of extinction in Canada, such analyses are challenging owing to a lack of comprehensive and accessible data reflecting information compiled from listing and recovery documents. To encourage ongoing synthesis and minimise duplication of efforts, we initiated CAN-SAR: a database of Canadian Species at Risk information. This transparent, open-access, and searchable database contains information transcribed from listing documents, including listing date, and derived variables. Derived variables required interpretation for which we developed standardised criteria to record information, including classification of recovery actions. The CAN-SAR database is updateable, and will contribute towards improved recovery planning to safeguard species of conservation concern.

## Abstract (summary)

**Table:** 

Measurement(s)	threatened species • threat classes • recovery actions
Technology Type(s)	document review
Sample Characteristic - Organism	multiple
Sample Characteristic - Location	Canada

## Background & Summary

Species that are in danger of extinction or extirpation have been identified and prioritized in lists compiled worldwide by national and international organizations^1^. In addition to extinction risk rankings, these lists generally include information on the species’ geographic range, population size, and threats that contribute to species declines. The International Union for Conservation of Nature (IUCN) Red List of Threatened Species is a well-known example of a species at risk list that provides a wide range of species information (http://www.iucnredlist.org)^2^.

Although the listing process focuses on individual species, once assembled, species at risk lists are powerful tools for biodiversity conservation and include key information that can inform conservation decisions and policies. Both national and global lists provide a valuable indicator of biodiversity^2,3^, present opportunities to synthesize information and trends related to, for example, the prevalence of particular threats^4–6^, and can be used to analyse listing and recovery processes^7,8^. However, species at risk lists can only achieve their full potential if the data informing them are made freely available in a standardized format that is easy to analyse and summarise^9,10^.

The Canadian Species at Risk Public Registry website (hereafter SAR Public Registry; https://www.canada.ca/en/environment-climate-change/services/species-risk-public-registry.html) contains the authoritative Canadian species at risk information, including Committee on the Status of Endangered Wildlife in Canada (COSEWIC) status reports (hereafter status reports), recovery documents, and listing decisions. However, there is no comprehensive publicly available database where researchers can easily access data appearing in various reports. There is a demonstrated need for this type of data as various studies have extracted similar information from status reports and recovery documents^5,7,9,11–20^ and recommended the creation of a database^9,21^. Such a database has the potential to maximise efficient use of scarce conservation resources by reducing duplication of efforts, and could amplify species at risk recovery efforts.

**Fig. 1:**
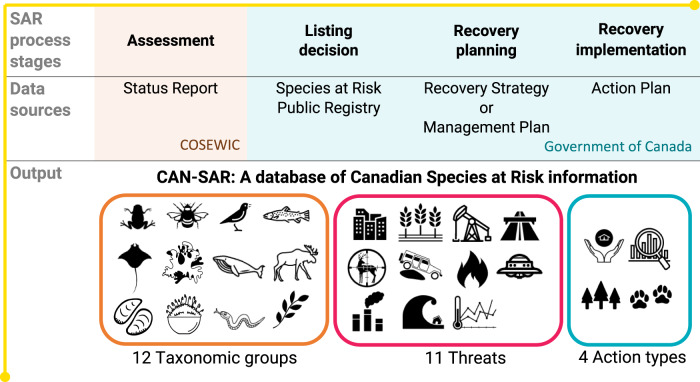
Data sources for CAN-SAR: a database of Canadian Species at Risk information. The database includes data extracted from multiple sources produced at various stages of the Canadian species at risk listing and recovery planning processes, including assessment, listing decision, recovery planning, and action planning. Primary responsible organizations at each stage include the Committee on the Status of Endangered Wildlife Species in Canada (COSEWIC) and the Government of Canada. The database includes information extracted from 1146 documents relating to 594 wildlife species representing a diverse set of data fields including taxonomic group, threat categories, and action types. Taxonomic groups included amphibians, arthropods, birds, fishes (freshwater), fishes (marine), lichens, mammals (marine), mammals (terrestrial), molluscs, mosses, reptiles, and vascular plants. Recovery action types include outreach and stewardship, research and monitoring, habitat management, and population management. The Level 1 IUCN threat categories are defined in Table 1.

**Fig. 2:**
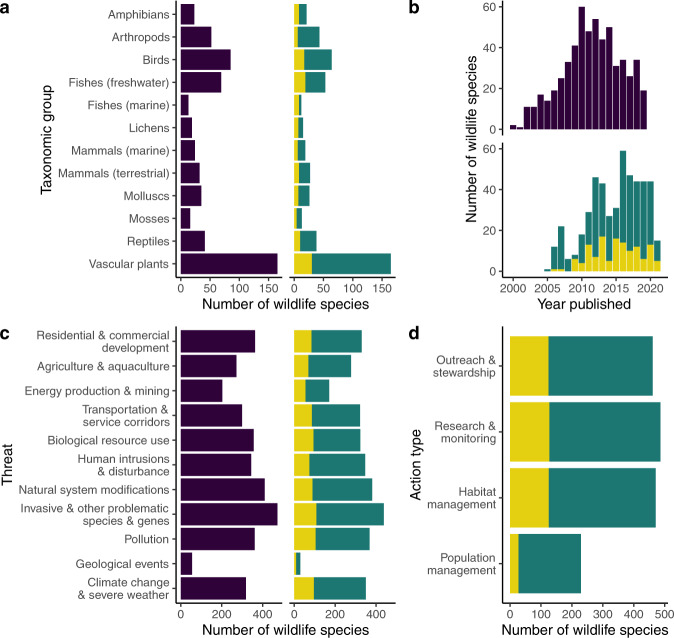
Overview of CAN-SAR: a database of Canadian Species at Risk information. The database includes data extracted from 1146 documents relating to 594 wildlife species (i.e. species or designatable units). Documents included status reports (purple), recovery strategies (green) or management plans (yellow). (**a**) Number of wildlife species with each document type included in the database classified into 12 taxonomic groups. (**b**) Distribution of publication dates by document type. (**c**) Prevalence of 11 IUCN Level 1 threat classes for wildlife species by document type. (**d**) Number of wildlife species where recovery documents specify each recovery action type (outreach and stewardship, research and monitoring, habitat management, and population management).

We introduce a resource intended to partially fill this gap; a Canadian Species at Risk (CAN-SAR) database^22^. We created the database by extracting information from publicly available documents for wildlife species legally listed under the Species at Risk Act (SARA) Schedule 1 as ‘Special concern’, ‘Threatened’, or ‘Endangered’ (Fig. 1). Extracted information is diverse and includes variables such as: assessed conservation rank, date of listing, various biological attributes, threats, recovery actions, and climate change specific information (Fig. 2). Our open-access database is designed to be updated with information on new wildlife species and variables of interest to the broader conservation research community. By building on this existing effort, researchers can make more efficient use of resources and avoid unnecessary duplication when performing analyses and syntheses related to Canadian listed species at risk. This is a new opportunity in Canada to explore a wide range of applied research questions concerning the recovery and protection of the nation’s biodiversity.

The CAN-SAR database has already been used to investigate factors that influence the treatment of climate change in Canadian extinction risk assessments and recovery plans^6^. Using data extracted from 510 status reports, Naujokaitis-Lewis *et al*.^6^ found that climate change was increasingly identified as a threat over time, however, few recovery documents specified climate-targeted recovery actions for climate-vulnerable species. This demonstrated the database’s value including suggested potential improvements associated with the integration of climate change considerations into current assessment and recovery planning processes.

The Canadian Wildlife Service (CWS) at Environment and Climate Change Canada is responsible for the overall administration and coordination of the SARA, and all data related to its implementation. CWS manages the authoritative source of data for species at risk in Canada and informs the public through the SAR Public Registry. Our vision of the CAN-SAR database complements existing efforts and aims to function as an open, accessible, comprehensive, living database^23^ that can be expanded upon as new data becomes available. Importantly, the CAN-SAR database is hosted on a searchable data repository and includes an accessible dataset that can be analysed across species, status reports and recovery documents, reflecting a powerful tool for synthesis. The SAR Public Registry is continually updated to reflect, for example, new listings and recovery documents. The CAN-SAR database is periodically updated and is formatted to facilitate analyses and synthesis of species at risk data. The CAN-SAR database can be accessed by a diversity of users to address salient applied research questions and can help pivot our knowledge of species at risk to action.

## Methods

The CAN-SAR^22^ database was created to provide access to publicly available data on species at risk in Canada in a standardized format that can be used in a wide range of applied research contexts. The variables included in the database were chosen to provide a range of information available for species at risk with a particular focus on climate change to support the first publication using the database^6^. The database includes numerous data fields including extinction risk status, various biological and geographical attributes, threat assessments, date of listing, recovery actions, and a set of climate change impact and adaptation variables. CAN-SAR is a living database that can be updated as new information and reports become available, or as other targeted data extraction efforts become available^23^.

In Canada, the listing process begins with an assessment of a wildlife species’ risk of extinction by the Committee on the Status of Endangered Wildlife in Canada (COSEWIC). A wildlife species can be either a species or a ‘designatable unit’, which includes subspecies, varieties, or other geographically or genetically distinct populations. Herein these are referred to collectively as ‘species’. COSEWIC is an independent body of experts who synthesize the best available information to date into a status report containing elements such as population size and trends, habitat availability, and threat assessments (Fig. 1)^17^. This report is then used as the basis for a status recommendation that is passed on to the Government of Canada, who makes the final decision on whether to legally list the species under Schedule 1 of SARA^24^. The species can be listed as ‘Special concern’, ‘Threatened’, ‘Endangered’, or ‘Extirpated’. If a species is listed as ‘Threatened’, ‘Endangered’ or ‘Extirpated’ then a recovery strategy is required, while for species listed as ‘Special concern’ a management plan must be created^24^. Recovery strategies must provide a description of the species’ needs, address identified threats, identify critical habitat (where applicable and to the extent possible), and include population and distribution objectives for the species’ recovery. Management plans include conservation measures for the species and its habitat^24^. Hereafter, we refer to recovery strategies and management plans collectively as ‘recovery documents’.

Information included in the database was extracted from various sources and documents that are available from the online SAR Public Registry, including COSEWIC status reports and status appraisal summaries, and recovery documents (Fig. 1). A COSEWIC status appraisal summary is produced instead of a new status report when a species has been previously assessed and COSEWIC experts are confident that its status will not change (https://www.cosewic.ca/index.php/en-ca/assessment-process/status-appraisal-summary-process.html). It is considered an addendum to the existing status report; thus, we use ‘status report’ to refer to either a status report or a status appraisal summary and the previous status report. From the SAR Public Registry website we accessed information from 1146 documents for all 594 species listed under SARA Schedule 1 as of March 23, 2021, that were classified with the status of ‘Special concern’, ‘Threatened’, or ‘Endangered’. Some species have multiple documents of the same type because COSEWIC reassesses at risk species every 10 years or less and recovery strategies and management plans are reviewed every 5 years and updated as needed. As new documents have become available they have been added to the CAN-SAR database without overriding the previously existing document, which allows for tracking of changes in various data fields over time. Only documents between 2018 and 2021, inclusive, have an updated version due to our updating schedule.

### Data extraction

Variables included in the CAN-SAR database were categorised as either directly transcribed or derived. Directly transcribed variables reflect information extracted from documents that require limited interpretation, such as scientific name or date of legal listing (Online-only Table 1). Derived variables reflect species’ attributes that required interpretation of text by data recorders (Online-only Table 1). The data dictionary (*CAN-SAR_data_dictionary.xlsx*) contains a description of each variable, including details of their extraction and synthesis^22^.

Several derived variables were extracted from the status report technical summary section, including whether the species is endemic to Canada or North America, and whether the species’ range is continuous with the United States. Endemism was determined for each species at two spatial extents, Canada and North America, based on descriptions of their global distributions from status reports. Whether a Canadian species’ range is continuous with its conspecifics in the United States was interpreted from descriptions of geographic isolation in the distribution and rescue effect sections of the status reports.

Variables related to species’ threats were derived from information in the status reports, recovery strategies and management plans. In 2012, COSEWIC initiated use of the IUCN threats classification system in status reports for some species; a ‘threats calculator’^25^. Threats calculators may also be included in recovery strategies and management plans. A threats calculator is a table included in the document that classifies threats into 11 general ‘level one’ classes and, more specific ‘level two’ subclasses (Table 1)^26^. Four variables (impact, severity, scope, and timing) for each level one and level two threats were scored independently and then combined into an overall impact score for each species. *Impact* is defined as the degree to which the species is threatened by the threat class; *severity* is the level of damage to the species from the threat class that is expected within ten years or three generations, whichever is longer; *scope* is the proportion of the species that is expected to be affected within ten years; and *timing* is the immediacy of the threat^25^. Threat-related variables were either transcribed directly from the threats calculator, or from the derived description of threats in the document if a threats calculator was not included.

**Table 1:** Definitions of level one threat classes and names of level two threat classes following Version 1.1 of the IUCN threats classification system.

Level 1 Threat Class	Definition	Level 2 Threat Classes
1. Residential and commercial development	Human settlements or other non-agricultural land uses with a substantial footprint.	1.1 Housing and urban areas
		1.2 Commercial and industrial areas
		1.3 Tourism and recreation areas
2. Agriculture and aquaculture	Threats from farming and ranching as a result of agricultural expansion and intensification, including silviculture, mariculture, and aquaculture.	2.1 Annual and perennial non-timber crops
		2.2 Wood and pulp plantations
		2.3 Livestock farming and ranching
		2.4 Marine and freshwater aquaculture
3. Energy production and mining	Threats from production of non-biological resources.	3.1 Oil and gas drilling
		3.2 Mining and quarrying
		3.3 Renewable energy
4. Transportation and service corridors	Threats from long, narrow transport corridors and the vehicles that use them including associated wildlife mortality.	4.1 Roads & Railroads
		4.2 Utility and service lines
		4.3 Shipping lanes
		4.4 Flight paths
5. Biological resource use	Threats from consumptive use of ‘wild’ biological resources including deliberate and unintentional harvesting effects; also persecution or control of specific species.	5.1 Hunting and collecting terrestrial animals
		5.2 Gathering terrestrial plants
		5.3 Logging and wood harvesting
		5.4 Fishing and harvesting aquatic resources
6. Human intrusions and disturbance	Threats from human activities that alter, destroy and disturb habitats and species associated with non-consumptive uses of biological resources.	6.1 Recreational activities
		6.2 War, Civil unrest and military exercises
		6.3 Work and other activities
7. Natural system modifications	Threats from actions that convert or degrade habitat in service of ‘managing’ natural or semi-natural systems, often to improve human welfare.	7.1 Fire and fire suppression
		7.2 Dams and water management/use
		7.3 Other ecosystem modifications
8. Invasive and other problematic species and genes	Threats from non-native and native plants, animals, pathogens/microbes, or genetic materials that have or are predicted to have harmful effects on biodiversity following their introduction, spread, and/or increase in abundance.	8.1 Invasive non-native/alien species
		8.2 Problematic native species
		8.3 Introduced genetic material
9. Pollution	Threats from introduction of exotic and/or excess materials or energy from point and non-point sources.	9.1 Household sewage and urban waste water
		9.2 Industrial and military effluents
		9.3 Agricultural and forestry effluents
		9.4 Garbage and solid waste
		9.5 Air-borne pollutants
		9.6 Excess energy
10. Geological events	Threats from catastrophic geological events.	10.1 Volcanoes
		10.2 Earthquakes/tsunamis
		10.3 Avalanches/landslides
11. Climate change and severe weather	Long-term climatic changes that may be linked to global warming and other severe climatic or weather events outside the natural range of variation that could wipe out a vulnerable species or habitat.	11.1 Habitat shifting and alteration
		11.2 Droughts
		11.3 Temperature extremes
		11.4 Storms flooding

Adapted from Salafsky *et al*.^26^.

For species where a threats calculator was included, we recorded whether each of the level one and level two threat classes were identified (i.e., considered a threat), and transcribed the scores for each of impact, scope, severity, and timing. Threat classes were considered identified if the impact was negligible, low, moderate, high, very high, unknown, or not calculated (outside assessment timeframe). Impact, scope, severity, and timing values were coded as ranked values of ‘0’: not a threat; ‘1’: neglible; ‘2’: low; ‘3’: moderate; ‘4’: high; ‘5’: very high; ‘-1’: unknown; ‘-2’: not calculated; or ‘NA’ where there were blank values. For exact ranking interpretations see *CAN-SAR_data_dictionary*^22^. For some species, the threats calculator was available from the COSEWIC Secretariat as a Microsoft Excel file, in which case threats information was extracted directly from the spreadsheet using R v 3.6.2^27^. For species where a Microsoft Excel file was not available, threats calculator information was manually extracted from the status report.

For species where a threats calculator was not included in the document, threats described in the text were classified into threat classes based on version 1.1 of the IUCN threats classification system (Table 1)^26^. Although a more recent version of the threats calculator exists, we applied version 1.1 classification to reflect the approach applied across the majority of species. Threats were considered identified if the threat was discussed as having any negative or potentially negative impact on the species. In cases where no threat calculator was available, the threat attributes of impact, scope, severity, and timing were scored as not applicable; ‘NA’.

Several variables were derived to determine how climate change was addressed in status reports and recovery documents. Whether climate change was mentioned anywhere in the status report was determined by searching the document for the words climat*, warm, temperat*, and drought. If a document contained any of these search terms, we assessed the context for description of anthropogenic climate change impacts. In cases where the terms were not found, the threats section was checked for any other descriptions that were related to climate change; if none were found, climate change was recorded as not mentioned. When climate change was mentioned, we then determined if it was identified as a threat by interpreting whether it was described as having a negative or potentially negative impact on the species. If a threats calculator was included in the status report, climate change was considered a threat if the ‘Climate change and severe weather’ threat class had an impact that was more than negligible or if climate change was described outside the threats calculator as a threat or potential threat. We recorded whether the threat of climate change was unknown. This included instances where climate change was described as having unknown effects on the species, if ‘unknown’ was assigned to impact, scope, severity, or timing in the threats calculator, or if knowledge gaps related to climate change were identified. Finally, the impact of climate change relative to other threats was classified based on descriptions of threats in the status report. The relative impact of climate change was classified as ‘0’ if it was not a threat; ‘1’ if it was described as a minor, potential, possible, or other threat; ‘2’ if it was a significant threat but not the most important or if it was among the list of threats with no indication of relative importance; or ‘3’ if it was among the most important threats described.

Additional derived variables extracted from recovery documents available on the SAR Public Registry included those related to critical habitat identification and recovery actions. For species with recovery strategies, we recorded whether critical habitat was described as identified, partially identified, or not identified. In cases where critical habitat was described as “identified to the extent possible”, it was marked as identified. We extracted information from recovery documents on what types of actions were recommended and whether the actions addressed the threat of climate change. Actions were categorized into four categories: outreach and stewardship, research and monitoring, habitat management, and population management (Table 2). Within each of the four categories, a set of 16 sub-types were recorded if any actions of that type were recommended or already completed. We also recorded action types and sub-types that specifically addressed climate change threats if climate change was listed as the threat addressed or the reason the action was necessary^6^.

**Table 2:** Categories of actions specified in Recovery Strategies.

Type of action	Action sub-type	Description
Outreach and stewardship	Education	Community outreach, stewardship, or education activities including providing information, hosting meetings, and coordinating with stakeholders.
	Mitigate climate change	Actions aimed at reducing or preventing greenhouse gas emission.
Research and monitoring	Monitoring	Gathering data to document trends over space and time, including population, harvesting, or habitat trends, and to assess effectiveness and track progress towards conservation and population objectives.
	Research	Producing new knowledge that contributes to improved understanding of a species, including but not limited to life history, population size and distribution, response to threats and response to actions.
Habitat management	Regulate human activities	Imposing restrictions on direct human threats such as changing logging, fishing or hunting rules, or legislation to manage other threats like pollution.
	Habitat restoration	Modifying habitat to improve suitability for species, including connectivity features like fish ladders and culverts.
	Invasive species removal	Removing invasive species that have negative impacts on species or habitats.
	Protection	Protecting habitat in any way including by zoning or creating protected areas. Including when a large portion of the population exists inside a protected area.
	Treat disease	Treating individuals or populations to prevent or treat disease.
	Manage native species negatively impacting species at risk	Relocating, culling, or excluding native predators including herbivores and cormorants.
Population management	Seed storage	Storing plant seeds in case of a need for reintroduction.
	Feeding	Artificially increasing the amount of prey or food available.
	Emergency response	Emergency measures to prevent extinction or extirpation, or prevent mortality by taking direct action.
	Captive breeding	*Ex situ* breeding to recover or maintain the population.
	Re-establishing populations	Artificially reintroducing or augmenting populations at inhabited or previously inhabited sites.
	Translocation	Moving species to previously unoccupied sites.

Recovery Strategies include recommended actions, actions in progress, and actions already completed.

Five data recorders conducted the initial data extraction, synthesis, and interpretation. All recorders were trained on the definitions, interpretation, and general process of data extraction to ensure consistent extraction of all variables. Data extraction occurred in multiple stages and included an iterative set of verifications and assessments of the same species among recorders to ensure consistent and standardized interpretations. Once convergence of interpretations was achieved, each recorder was assigned a set of species/reports from which to extract information.

### Next steps

The CAN-SAR database is intended to be a living database that can be updated by adding information from new documents or species as they become available, adding more historical documents, or extracting new information from all documents. The current set of species and associated information includes those listed on Schedule 1 of SARA (as of March 23^rd^ 2021) as ‘Special concern’, ‘Threatened’, or ‘Endangered’. Examples of future data additions include integration of data from species assessed by COSEWIC that are not listed under Schedule 1 of SARA, adding fields that specify the criteria used to arrive at a risk status designation, and integration of data from action plans. We anticipate updating the database periodically, as time and resources allow, and we also encourage anyone interested in extending or expanding on the CAN-SAR database to communicate to discuss a collaboration. Integration of new datasets will require screening and validation to ensure adherence to data standards and consistent interpretations. In the longer term, we foresee the implementation of automatic updating of the CAN-SAR database for variables that do not require interpretation by using machine-readable formatted status and recovery documents.

### Applications

Applications of the CAN-SAR database reflect both opportunities to synthesise the data in novel ways and to expand the scope of the current database to include new data fields representing information contained in status assessments and recovery documents. The CAN-SAR database facilitates independent data analysis and synthesis efforts ranging from trend analysis of threats, identifying research and monitoring gaps, and assessing the effectiveness of recovery actions, which target various steps of the listing and recovery process. For example, the database provides a platform to extend existing climate change focused work^6^ to assess the prevalence of recommended climate change targeted recovery actions, such as translocations. With recent adoption of the ‘Pan-Canadian approach to transforming Species at Risk conservation in Canada’^28^, which emphasizes multi-species recovery planning approaches, there is an opportunity to assess patterns in key sectors, which include agriculture, forestry, and urban development, over time and by taxa and how they map to threats.

With the integration of additional variables through future data extraction or integration efforts, the CAN-SAR database can be used to assess novel questions. For example, broadening recovery action categories to include those that reflect natural climate solutions can highlight where recovery efforts may provide co-benefits, thus achieving biodiversity conservation and climate change mitigation goals^29^. Specifically, habitat restoration actions for a forest-dependent species primarily threatened by habitat loss may lead to improved recovery outcomes while also resulting in carbon sequestration and improved climate change mitigation efforts. Tracking these types of actions in CAN-SAR could highlight both opportunities and gaps for the integration of climate smart conservation principles^30^ into species at risk recovery planning and the adoption of climate change adaption measures for species directly considered climate change threatened and those that are not^6^.

## Data Records

The CAN-SAR database is provided as a .csv  file (*CAN-SAR_database.csv)* that is available from the Open Science Framework^22^. Each row in the database represents a single document and species or designatable unit and columns include information for each variable described above (Online-only Table 1). A data dictionary is provided as a Microsoft Excel workbook (*CAN-SAR_data_dictionary.xlxs)* and is available from the Open Science Framework^22^. The data dictionary contains four spreadsheets: 1) *Introduction* includes citations and acronyms contained in the database; 2) *Database Fields* includes a description of each field (or variable) in the database, how it was extracted and the format of the data; 3) *Action Types* includes a description of each action category and sub-category as outlined in Table 2; and 4) *Threat Classes* includes a copy of version 1.1 of the IUCN threats classification system. Machine-readable metadata is also provided in the Ecological Metadata Language^31^ (*eml.xml*). This metadata was produced from the data dictionary using the EML R package^32^.

## Technical Validation

In addition to the internal consistency checks applied during the data extraction phase, we independently validated the dataset to determine an overall level of agreement between original data sources and the database. This validation was performed on the 533 species with documents published before February 22, 2018 and included all level 1 threats classes extracted from status assessments. The validation spreadsheet included all variables from the CAN-SAR database except for those transcribed from the SAR Public Registry website and those related to actions recommended in the recovery document. The recovery action variables were not included in the validation because they were assessed after the other variables in the database by the same data recorder who did the validation. Variables that were directly transcribed from the data source to the database (Online-only Table 1) were left in place and checked against the information in the data source. All other variables were blank in the validation spreadsheet and were re-extracted without reference to the original dataset. Directly transcribed variables were checked rather than re-extracted because they are not subjective, so the extractor was not influenced by seeing the original values. We performed data validation separately for species with and without a source threats calculator. Because the classification into the 11 threat classes was done by the status report writers and not the data recorders, we expected threats information extracted from the threats calculator to be more consistent.

We randomly selected 10 out of the 104 species (i.e., approximately 10% of records) where a threats calculator was available for validation. Overall, there was 3.1% disagreement between the validation and original data. Eighty-nine of the 97 cases of disagreement were in threat-related fields for which there was 3.5% disagreement. The remaining fields disagreed only 1.5% of the time.

We randomly selected 41 out of the 406 species (i.e., approximately 10% of records) with no threats calculator available for validation. Overall, there was 4.3% disagreement between the validation and original data. For the threat-related fields, there was 11.8% disagreement and for the remaining fields there was 2.8% disagreement.

Our validation process showed that the level of disagreement amongst data recorders were sufficiently low overall. We found greater differences among data recorders for species with no threats calculator. This was expected; as other studies have noted, threat classes are not mutually exclusive and interpretation is part of assigning threats to a class^33^. A preliminary (and ongoing) data validation of data entered reflecting 2018–2021 time frame suggests that error rates are lower across all fields, when compared to earlier records ( < 2018).

We created an automated process using continuous integration to test the internal consistency of the database. Continuous integration allows the automatic testing of data or code whenever any changes to the database occur, allowing for updated real-time quality control^23^. We used the continuous integration system offered by GitHub called ‘GitHub Actions’. Whenever changes are made to the GitHub repository that holds the data, a GitHub Action is triggered which runs an R script to test the internal consistency of the data. If errors are found in the data a notification is sent to the database maintainer allowing the error to be fixed. The data is checked for missing values in required fields, that data is of the correct type and has values within the expected range and that the data is internally consistent. For example, if climate change is identified as a threat the relative impact of climate change must be greater than zero.

In sum, open, accessible, and timely information on species at risk will allow for independent analyses and data syntheses that will contribute to effective recovery planning. The CAN-SAR database reflects initial efforts at making such data available to scientists in an aim to minimise the duplication of efforts and encourage future collective data syntheses.

## Data Availability

The code used to test the internal consistency of the database is publicly available at the GitHub repository https://github.com/LandSciTech/CANSARD.

## Online-only Table

**Table:** Online-only Table 1 Variables and the original data source included in CAN-SAR: a database of Canadian Species at Risk information.

Variable	Source	Type of variable
Common name	Status Report, Recovery Strategy or Management Plan	Directly transcribed
Species	Status Report, Recovery Strategy or Management Plan	Directly transcribed
Taxonomic group	Status Report, Recovery Strategy or Management Plan	Directly transcribed
Document type	Status Report, Recovery Strategy or Management Plan	Directly transcribed
SARA status	SAR Public Registry	Directly transcribed
Document citation	Status Report, Recovery Strategy or Management Plan	Directly transcribed
Date published online	SAR Public Registry	Directly transcribed
Publication year	Status Report, Recovery Strategy or Management Plan	Directly transcribed
Whether the document is a status appraisal summary	Status Report, Recovery Strategy or Management Plan	Directly transcribed
Whether the document is final	Status Report, Recovery Strategy or Management Plan	Directly transcribed
Whether the document is an amendment	Status Report, Recovery Strategy or Management Plan	Directly transcribed
Whether a threats calculator was included	Status Report, Recovery Strategy or Management Plan	Directly transcribed
Threats calculator version	Status Report, Recovery Strategy or Management Plan	Directly transcribed
Threats calculator date	Status Report, Recovery Strategy or Management Plan	Directly transcribed
Threats calculator assessors	Status Report, Recovery Strategy or Management Plan	Directly transcribed
Threats calculator references	Status Report, Recovery Strategy or Management Plan	Directly transcribed
Threats calculator calculated overall impact	Status Report, Recovery Strategy or Management Plan	Directly transcribed
Threats calculator assigned overall impact	Status Report, Recovery Strategy or Management Plan	Directly transcribed
Threats calculator impact adjustment reasons	Status Report, Recovery Strategy or Management Plan	Directly transcribed
Threats calculator overall comments	Status Report, Recovery Strategy or Management Plan	Directly transcribed
COSEWIC status	Status Report	Directly transcribed
Presence or absence in each province, territory or ocean	Status Report	Directly transcribed
Estimated extent of occurrence	Status Report	Directly transcribed
Index of area of occupancy	Status Report	Directly transcribed
Locations	Status Report	Directly transcribed
Status Report writers	Status Report	Directly transcribed
Whether the species is endemic to North America or Canada	Status Report	Derived
Whether the species’ range is continuous with the United States	Status Report	Derived
Level one threats identified	Status Report, Recovery Strategy or Management Plan	Derived
Level two threats identified	Status Report, Recovery Strategy or Management Plan	Derived
Impact, severity, scope and timing scores for level one and two threats	Status Report, Recovery Strategy or Management Plan	Derived
Whether climate change was mentioned	Status Report, Recovery Strategy or Management Plan	Derived
Whether climate change was identified as a threat	Status Report, Recovery Strategy or Management Plan	Derived
Whether the threat of climate change was unknown	Status Report, Recovery Strategy or Management Plan	Derived
Whether climate change knowledge gaps were identified	Status Report, Recovery Strategy or Management Plan	Derived
Whether climate change had unknown impact, scope, severity or timing	Status Report, Recovery Strategy or Management Plan	Derived
Relative impact of climate change	Status Report, Recovery Strategy or Management Plan	Derived
Date COSEWIC assessment was completed	SAR Public Registry	Directly transcribed
Date species added to SARA Schedule 1	SAR Public Registry	Directly transcribed
Decision of the Governor in Council	SAR Public Registry	Directly transcribed
Whether critical habitat was described as identified	Recovery strategy	Derived
Action types	Recovery strategy or Management Plan	Derived
Action sub-types	Recovery strategy or Management Plan	Derived
Whether any actions addressed the threat of climate change	Recovery strategy or Management Plan	Derived
Action types that addressed the threat of climate change	Recovery strategy or Management Plan	Derived
Action sub-types that addressed the threat of climate change	Recovery strategy or Management Plan	Derived

Variables were categorised as one of ‘directly transcribed’ or ‘derived’. Directly transcribed variables reflected information extracted directly from the data source, whereas derived variables required interpretation, based on clearly defined standards and definitions, by the data recorder. SAR Public Registry and reports: https://www.canada.ca/en/environment-climate-change/services/species-risk-public-registry.html. Estimated extent of occurrence, index of area of occupancy and locations are defined in https://www.cosewic.ca/index.php/en-ca/about-us/definitions-abbreviations.html.
